# Pragmatic progress? Trade-offs and seed choices in Tajikistan

**DOI:** 10.1007/s10460-026-10855-z

**Published:** 2026-04-15

**Authors:** Michael Spies, Irna Hofman

**Affiliations:** 1 Leibniz Centre for Agricultural Landscape Research (ZALF), Müncheberg, Germany; 3 Eberswalde University for Sustainable Development, Eberswalde, Germany; 2 University of Oxford, Oxford, United Kingdom; 4 Leiden University, Leiden, The Netherlands

**Keywords:** Seed, Tajikistan, Paradigms, Socio-technical regime, Political ecology

## Abstract

Seed features prominently in the polarised academic and policy debates about the future of agriculture. In this article, we examine maize and cotton farmers’ seed decisions in Tajikistan, where the Soviet-era ideal of industrial modes of agriculture continues to influence farming practices, but where interventions by international actors, along with new paradigms, increasingly affect seed systems. What factors affect farmers’ seed choices, and what notions of agricultural progress do their strategies reveal? Based on extensive fieldwork in lowland Tajikistan, we examine these questions, drawing on political ecology and transition studies scholarship. We consider the state, the market, technology, and crop characteristics and illuminate starkly contrasting seed preferences for maize and cotton. Farmers opt for high-yielding hybrids for the former and relatively low-quality local seed for the latter. In order to explain this apparent inconsistency in seed choices, we address the importance of attending to the context shaping farmers’ seed decisions. These choices reflect pragmatic rather than paradigmatic strategies and challenge presumed dichotomies of distinct development pathways associated with local versus improved seed.

## Introduction

Seed features prominently in academic and policy debates about the future of agriculture. On the one hand, proponents of technology-intensive, productivist modes of agriculture argue for advanced plant breeding as key to ensure food security for a growing world population, and as a panacea for agricultural woes, also in light of climate change’s impact on agriculture (see, e.g., Levidow et al. 2014; Wilson and Burton 2015; OECD 2018; Qaim 2020). On the other hand, advocates of more localised and low-input farming approaches point to the importance of (agro)biodiversity, local knowledge, farmers’ autonomy, and ecological resilience for sustainable agrarian futures. They criticise technology-intensive approaches for the rapid spread of “modern” or “improved” seed varieties that may threaten resilient, autonomous farming and ecosystems, and reinforce the concentration of market power (Kloppenburg 2004; Luby and Goldman 2016; Schapiro and Talbot 2018; Sievers-Glotzbach and Christinck 2021; Sandström et al. 2024). However, proponents of both paradigms and pathways have been found guilty of leaning on dogmatic and essentialised viewpoints (Luna 2020; Curry 2023).

Thus, the debate on seeds, as part of broader discussions on the future of agriculture, is polarised. The debate departs from a contrast between production and profit maximisation and control over resources. A case in point is hybrid seed: based on an advanced breeding technology of combining desired traits from two parental lines in one crop variety, hybrid seeds have been a huge commercial success, among other aspects due to their high yields, high plant uniformity, and their “‘natural’ intellectual property protection” (ter Steeg et al. 2022, p. 463) of not reproducing true to type. However, the debate goes beyond hybrid varieties alone; it concerns the merits and threats of “improved” seeds more broadly, i.e. seed varieties developed through scientific and commercial breeding that is fundamentally different from the development and maintenance of, and the sociopolitical organisation involved in, local landrace varieties (see Mueller and Flachs 2022).^1^ As such, the promotion and use of improved seeds is associated with a productivist paradigm, whereas proponents of extensive farming models or pathways typically promote local seeds. 

The critical agrarian scholarship sheds light on the complex factors affecting farmers’ seed decisions (see Kilwinger et al. 2020; Arora et al. 2024). However, the nuanced and plural perspectives of agricultural producers often receive little attention in the debates on seed choices and production strategies, including “farmers’ complex opinions in their own words” (Mueller and Flachs 2022, 456). There is a need to focus on farmers and on the complex sociopolitical, economic, ecological and technological environments in which their decision-making takes place.

Aiming to contribute to the literature on seed politics and agricultural knowledge (Flachs and Stone 2019; Kilwinger et al. 2020; Luna 2020; Curry 2023; Arora et al. 2024), we analyse seed system dynamics and the seed choices of maize and cotton producing farmers in Tajikistan. Whereas both cotton and maize are cash crops, signified by commodified supply chains,^2^ we observe a stark contrast in seed choices, which warrants attention. Seeking to understand and explain this contrast, we examine, firstly: What are the primary factors that shape Tajik farmers’ seed choices? Secondly: What strategies do farmers pursue? Lastly: What notions of agricultural progress do these strategies reveal? We draw on qualitative, multi-sited research independently undertaken by both authors in lowland Tajikistan (primarily) between 2021 and 2023. We engage the scholarship on socio-technical regimes (Geels 2011, 2019); political ecology (Lawhon and Murphy 2012); and, recent studies on (constrained) imaginaries (Schmook et al. 2023). This interdisciplinary lens enables us to identify and incorporate a complex set of factors and dimensions affecting Tajik farmers’ seed choices. The appraisal of seed differs across farming populations, where ecological systems, farmers’ and state priorities, the availability of technology, and the characteristics of outlets condition farmers’ seed practices (Arora et al. 2024; Flachs and Stone 2019; Kilwinger et al. 2020).

Tajikistan offers an insightful case for critical agrarian scholarship and studies of seed politics. Seed policies and imaginaries put forward by the Tajik state and (most) domestic agricultural specialists align with the productivist paradigm found in the international debate. However, this productivist ideology has not been introduced recently in Tajikistan. It has its roots in the Soviet era. At the same time, Tajikistan’s agricultural sector relies to a significant extent on foreign donor aid and investment. In recent years, diverse foreign actors have propagated specific visions, ideals, and models of agricultural development. Notably, the transnational corporate actors that dominate the global seed regime, and whose concentration of power is at the centre of much critique (on these actors, see ETC Group 2022; Clapp 2021) do not play a major role in Tajikistan. However, the proliferation of commercial and mainly imported improved seed varieties has progressed in recent years. Thus, various visions (or models) of agricultural development are promoted in Tajikistan. The context of Tajikistan is unique in another respect in that agriculture is performed in a context in which the authoritarian state exerts considerable influence on farmers’ production practices. It attempts to control land use patterns in various ways. In later sections, we shed light on differences in the politics of maize and cotton production, and analyse the role of production politics in seed choices. Indeed, farmers are situated in a complex sociopolitical environment that is marked by the Soviet legacy, but is highly dynamic at the same time. 

Our article is structured as follows. In the next section, we provide the conceptual framework of our analysis, focused on the notion of the socio-technical regime and political ecology. A brief, third section presents the research methods, followed by section four, which introduces the context of Tajikistan. Section five is the empirical heart of our paper, in which we analyse cotton and maize seed use in Tajikistan. We discuss our findings in section six, followed by a concise conclusion. Our findings illuminate the importance of a context-specific analysis to understand Tajik farmers’ seed choices. Rather than paradigms, farmers’ decisions reflect pragmatism. They challenge the dichotomy of pathways associated with local versus improved seed.

## Analytical lenses to understand seed choices

Understanding farmers’ seed choice is complex. Seed systems are embedded in a broad, multi-dimensional environment. Seed choices result from the interaction between a complex set of political, technological, socio-cultural, economic, and ecological factors. Within the set of structures, farmers enjoy agency that shapes their practices. Seed practices can change or remain stable over time as seed is reproduced, saved and (incrementally) improved by incumbent actors, such as firms, engineers, users, policy-makers and regulators, and special-interest groups (Mueller and Flachs 2022).

The academic literature on seed choices and seed politics is diverse, and analytical lenses vary, from critical political economy and agrarian studies (Luna 2020; Taylor 2020), theories of commodification and agricultural knowledge (Flachs and Stone 2019; Amanor 2024) to science and technology studies (Almekinders et al. 2019; Kilwinger et al. 2020). In order to analyse and appreciate the complexity involved in seed choices, we draw on the notion of the socio-technical regime (Hebinck 2001; Geels and Schot 2007; Geels 2011) and political ecology scholarship (Lawhon and Murphy 2012; Sinha 2022). Bridging these bodies of scholarship enables us to incorporate various factors and dimensions of seed choice, including the role of markets, the state and governance more generally, the politics of production, and the commodification of seed (Flachs and Stone, 2019).

The notion of the socio-technical regime has been used to make explicit the role of various actors including scientists, policy makers, users and technology in patterning processes of technological change; they explicitly or implicitly act according to specific rules and norms, being embedded in “institutions and infrastructures” (Rip and Kemp 1998, p. 338). In the context of agriculture, farmers are active constituents of a socio-technological regime (Hebinck 2001). Their seed decisions can be considered “socio-technical practices” (Lawhon and Murphy 2012, p. 358).

The socio-technical regime offers a perspective, as Hebinck (2001, 120) notes in his study of hybrid maize in Kenya, “capable of handling, at the same time, the artefacts, the designers as well as the so-called ‘end users’ of technology, as well as the social interactions between them.” Thus, it can be understood as comprised of different, albeit interdependent, actors and artefacts, and the rules they implicitly or explicitly prescribe.^3^ The regime is situated in a “broader socio-technical and institutional context”, also referred to as landscape, which “involves policy discourses of the state such as technological progress and modernisation that target the further development of specific technology trajectories […]” (Hebinck 2001, p. 125). The relative influence and capacity of the various actors and artefacts involved differ. Indeed, power dynamics are inherent in the socio-technical regime.

Within the socio-technical regime, Geels (2011) identifies five dimensions or subregimes: the technological regime; the science regime; the policy regime; the socio-cultural regime; and, the user and market regime.^4^ We add an explicit political ecology lens for our analysis, drawing on Lawhon and Murphy (2012). We do so in two ways. First, we consider more explicitly “the social processes and power relations through which knowledge and technologies are created, transformed, and shared” (Lawhon and Murphy 2012, p. 365). In doing so, we draw clear links between Geels’ (2011) subregimes. Second, we take more explicitly into account the material dimension of seed choices – including biophysical crop properties, as “the properties of certain [seed] technologies shape the way in which they become embedded” (Lawhon and Murphy 2012, p. 368). Thereby we also respond to recent calls for a political ecology of crops that draws attention to their political dimension while also considering “crops as co-producers of agrarian change” (Fischer et al. 2022, p. 92). With this diverse body of scholarship, we aim to avoid the pitfall of technological determinism on the one hand or solely focussing on power and social processes on the other.

Against the background of these interacting forces and actors, attempts to introduce new technologies and agricultural inputs, including seed, are not necessarily successful (Kilwinger et al. 2020). Indeed, seeds do not always land on fertile soil (see also Hofman 2024). What is more, some factors influence farmers’ practices and seed choices directly, while others do so in more indirect, complex ways. For instance, by influencing farmers’ perceived ability to pursue specific practices or goals, and their vision of progress, external actors can indirectly affect farmers’ seed decisions.

The latter speaks to the notion of “constrained imaginaries” (Schmook et al. 2023). Seed decisions may be made instantly, but they are also influenced by imaginaries, i.e. (shared) visions or ideas of a desired condition or pathway of progress. As such, farmers’ imaginaries are constrained by material realities, as well as by dominant paradigms and thus by “what farmers perceive as possible and not possible” (Schmook et al. 2023, p. 311; see also Zuberi et al. 2025). Indeed, it is difficult to envision farming “otherwise” when one is surrounded by and depending on service-providers, guided by policy-makers, informed by agricultural scientists, and pushed into certain directions by market actors. Off-farm actors’ notions and ideologies can, directly (by prescribing) or indirectly, narrow down directions of progress (see also Flachs et al. 2025). Thus, farmers’ seed choices do not necessarily reveal their own imaginaries, which Schmook et al.’s (2023) analytical lens can explain. Thus, the processes shaping farmers’ seed practices are complex.

In what follows, we first describe our research methods, before presenting the context of Tajikistan and our case studies.

## Research methods

This article draws on qualitative research data collected by both authors during individual fieldwork in lowland areas of Tajikistan, predominantly the southwestern Khatlon region, between 2020 and 2023. It also builds on earlier research of Hofman (briefly detailed below). While we worked independently, engaged in two different research projects, we were in touch during and between field research. Our regular exchange of insights and reflections culminated in this joint writing. 

Michael Spies conducted four months of field research in Tajikistan in 2022 and 2023, examining seed system changes with a focus on maize. The objective of the research was to gain a deeper understanding of the drivers and consequences of the recent shift from open pollinated to hybrid maize varieties imported from China, primarily through qualitative interviews with various seed system actors. Research was conducted in collaboration with local research assistants. A total of around 60 interlocutors were interviewed, including 21 farmers operating small to medium-sized farms of approx. 2–15 hectares, mostly managed as family enterprise. A few of them were managed jointly with one or two other farm households. In addition, around 20 seed dealers, breeders, state officials (at various levels of the state administration), processing companies, as well as representatives of large farm enterprises and foreign organisations involved in seed distribution were interviewed. Besides interviews, informal conversations with these seed system actors and observations during field visits provided additional insights. The primary data informing the first case study of this article results from the interviews with farmers, which aimed at gaining a comprehensive understanding of their perspectives and motivations on seed decision-making.

The second case study primarily draws on data collected by Irna Hofman during a fieldwork period of 20 months between 2020 and 2021, with a few follow-up interviews in 2022 (by phone) and 2023 (in person and by phone). Hofman examined cotton seed system dynamics as part of longitudinal research on the political economy of agrarian change. Research was undertaken in various parts of lowland Tajikistan, with a primary focus on the Khatlon region. As part of multi-sited ethnographic research in rural Tajikistan, Hofman undertook 38 interviews with farmers specifically focused on seed choices (various farm sizes, mostly 2–15 hectares), and 34 semi-structured interviews with various actors, including state officials working at multiple levels of the state administration, seed specialists and scientists. In addition, together with two research assistants, a survey was carried out in February 2021 (*N* = 233, of which 155 farmers were engaged in cotton farming). The questionnaire included questions on seed choice (see also Hofman 2024). Whereas this article does not refer to the survey data explicitly, the findings have provided contextual insights on trends and factors affecting seed choice. During fieldwork between 2020 and 2021, Hofman also visited three of the four research stations of the Tajik Academy of Agricultural Sciences and held four interviews with employees of seed breeding farms orientated towards cotton seed breeding. In addition, she participated in a public event focused on the seed sector in January 2021. During a short visit to Tajikistan in August-September 2023, Hofman interviewed five cotton farmers. The analysis of Hofman also builds on dissertation research, for which fieldwork was undertaken in various phases between 2012 and 2015.

We are cognisant of the fact that farmers’ attributes and differences in terms of social strata, gender, and generation, as well as farm size affect their decision-making (see, for instance, Kilwinger et al. 2020; Arora et al. 2024). However, in this article, we mostly refer to farmers in aggregate, not differentiating legal status, scale/farm size, or other characteristics – unless these factors make a difference for their seed choices. As described below, we observed that attention to social differentiation is important for understanding cotton seed choices. In the following sections, we go down to Tajikistan’s countryside, where we analyse the various actors and factors affecting farmers’ seed choices.

## The context of Tajikistan

Tajikistan is a small landlocked country in Central Asia with a population of over 10 million people. Due to its mountainous terrain, crop production is largely concentrated in the lowlands of Khatlon in the southwest, the lowland areas in the localities known as “the Districts of Republican Subordination” that surround the capital city of Dushanbe, and the Sughd region in the northwest. Due to the semi-arid to arid climate in these areas, crop production relies on irrigation made possible through a vast network of canals and pumping stations carrying water from glacier-fed rivers to the fields. This concerns mostly gravity-fed irrigation. In hilly and mountainous areas, rainfed agriculture is practiced. In this article, we focus on agricultural producers in irrigated areas. 

During the Soviet era, the state played a central role in the research, breeding, and supply of seeds and seedlings of plants that were of vital importance to the economy: cotton, as well as wheat and tobacco. The Soviet ideal of industrialised farming resulted in large-scale farming systems, monoculture cropping, intensive use of agro-chemicals, and efforts to mechanise the production as much as possible. Production was meticulously monitored and controlled, although local-level farm leadership could influence decisions stipulated at the top.^5^

The break-up of the Soviet Union had a significant impact on Tajikistan’s seed systems. The ending of subsidies from Moscow significantly affected the centralised supply channels of agricultural inputs, and Tajikistan’s civil war that followed soon after independence (1992–1997) deepened the impact. At the same time, with independence, foreign inputs, knowledge, and ideologies from countries beyond the former Soviet realm started to enter more profoundly (than before). Whereas agricultural reforms set in motion in the 1990s were expected to end the planned economy, for the state’s strategic crops, particularly cotton, the state has attempted to retain control over production, including over cotton seed circulation and sales. Further, a constant thread throughout Tajikistan’s post-socialist period has been the continuation of a productivist agricultural development paradigm. It is a legacy of Soviet agriculture, that marks agricultural policies, ideologies, and practices.

Farmland is state-owned in Tajikistan. Agricultural producers can access land under different tenure relations.^6^ State ownership of land puts agricultural producers at risk of arbitrary decisions by local authorities, as the state can revoke land usage rights in case of ‘inappropriate’ land use, which includes leaving land fallow for too long or refusing to produce specific crops (Hayward et al. 2022). In this way, the country’s autocratic regime maintains a strong hold on agricultural production, incentivising and sometimes forcing farmers to grow specific crops – in particular cotton, the country’s primary strategic crop for export (Hofman and Visser 2021). Other important crops include potato alongside wheat as the main staples, maize produced as fodder for livestock, and various fruits and vegetables. The primary agricultural producers are commercially-orientated, so-called *dehqon* farms that have been formed since the 1990s, when land reforms were initiated to dissolve the collective and state farms after the break-up of the Soviet Union (Lerman and Sedik 2018). Farm restructuring has been continuing ever since, resulting in a large variety of agricultural producers. *Dehqon* farms are diverse, in terms of ownership, size (from less than one to over 100 hectares), and cropping patterns. Many are managed as private (family) farms, with (often nominal) shareholders (Mukhamedova and Wegerich 2018; Hayward et al. 2022). In addition to *dehqon* farms, there are production cooperatives and large farm enterprises or agribusinesses with land holdings of sometimes several hundred or even thousands of hectares. Thus, the population of agricultural producers is anything but homogeneous,^7^ and differences in social, political, and financial capital have a strong bearing on farmers’ agricultural practices. For instance, farmers with good connections to state officials can negotiate cropping patterns, whilst others invest their political capital to access quality agricultural inputs (Hofman and Visser 2021). Exemplary, for instance, is that large elite-run enterprises enjoy preferential treatment in terms of access to critical resources such as water (Nekbakhtshoev and Babu 2022). Agricultural producers are also diverse in terms of age and training. Many elderly farmers came of age and gained skills as labourers of Soviet large-scale farms. The younger generation of farmers moved more recently into farming, often as a result of inheritance of land. Many of them rely on senior agronomists, older farmers, or private sector extension agents.^8^ Thus, the context is highly dynamic, with ongoing changes in farming structures and the characteristics of agricultural producers.

When it comes to seed, the Tajik state promotes the import of seed, and also supports the growth of private seed business, so as to enhance productivity. The domestic seed breeding sector has become impoverished after the break-up of the Soviet Union, to a large extent as a result of the lack of state support. State officials express a wish to join the International Union for the Protection of New Varieties of Plants (UPOV). In their view, it could offer a great incentive for Tajik and foreign seed breeders to expand their activities in the country. Their intentions regarding commercial seed breeding are also encouraged by international organisations. Indeed, ideas about the best pathway of agricultural development in Tajikistan have been influenced by external, i.e. foreign actors and the plethora of actors engaged in Tajikistan’s rural economy has expanded in recent years. It comprises actors promoting industrial agriculture (e.g., the World Bank, and, until recently, USAID) as well as non-governmental organisations advising more localised farming systems (e.g., the German organisations GIZ and Welthungerhilfe). As a case in point, the World Bank’s International Finance Corporation set up an agricultural extension agency in the early 2000s, which later became the official distributor of Syngenta in Tajikistan. In what follows, we examine: What seed choices do cotton and maize growing farmers make, and why?

## Unpacking seed decisions

### Dynamism: The proliferation of hybrid maize

Produced as fodder for their own livestock, but also for sale on the local market, maize is typically cultivated by farmers in Tajikistan’s lowlands as a first crop or late summer crop after harvesting wheat. With cotton being the priority crop, maize is usually produced on a relatively small share of not more than 10–20% of farmers’ land. Maize fields of more than one hectare are exceptional.

Over the last decade, a major shift has occurred in the maize sector in Tajikistan: Local open-pollinated varieties (OPVs), which have been traditionally sown since Soviet times, have been largely replaced by hybrid varieties, (predominantly) imported from neighbouring China. These seeds have proliferated across the main production areas of lowland Tajikistan where they now dominate maize fields. Out of the 21 interviewed farmers located across Tajikistan’s lowlands in 2023, 19 had shifted to hybrid maize seeds, mostly within the last five years. Only two were still sowing OPVs. The shift towards hybrid maize is salient, and is observed not only in the fields but also on the seed markets in cities and towns: Spies visited more than 20 seed shops and seed stalls across the country’s three primary agricultural regions, and observed that hybrid maize seed dominated in the vast majority of the outlets.

A primary reason for choosing hybrid seems to be the yield increase. Reportedly, compared with OPVs, significant yield increases can be achieved by using these hybrids: interviewed farmers reported yields of up to 10 tons per hectare from hybrid seeds, sometimes even more, whereas the two farmers using OPVs reported four to five tons (see also Zakirova et al. 2023). Indeed, many respondents explicitly mentioned yield as the reason for choosing hybrids: hybrids performed better, compared with previously sown OPVs. As a farmer stated: “People were not happy with [OPVs]. The plants just grew tall, but did not produce any cob” (interview, 24 March 2023). However, the profits of sowing high yielding hybrids depend not only on yield: the net benefit depends on production costs, including expenses related to seed. The latter are relatively high for hybrids as farmers need to buy them seasonally. At the same time, the market price for harvested hybrid maize grains tends to be lower than for OPVs. When asked about other input requirements, in particular water and fertilisers, farmers’ responses did not reveal any significant differences between hybrids and OPVs. However, according to an interviewed seed expert, the hybrid varieties are more responsive to fertilisers and yield can increase up to 18–20 tons per hectare with optimised fertiliser use.

As such, a calculation of net benefits needs to consider various factors, and farmers’ seed choices cannot be reduced to yield expectations alone. Beyond market prices and production costs, there are other factors of importance. Some farmers believe that the Chinese hybrids are more pest-resistant than local varieties due to chemical pre-treatment of seeds. Moreover, some farmers hold that imported seeds, usually sold in small 2.5 kg bags, are superior and more reliable than locally sold OPVs. We observed distrust of seed dealers: some farmers perceive packaged seeds more trustworthy than unpackaged ones, due to experiences with seed being tampered with by producers or dealers. As one farmer stated: “People cheat! [It is] better to use the packed hybrid ones, we can rely on them” (31 March 2023, see also Spies 2025). Furthermore, several farmers and specialists question the technical capacity of domestic seed breeders and consider imported varieties of higher quality. As a result, as a leading seed expert in the country summed up: “People believe in the superiority of imported seeds” (21 April 2022). Many state officials also look to foreign companies and organisations for seed.

Having said that, not everyone shares such convictions. Some farmers deliberately choose to sow OPV of maize. As one of them explained:

Since [my] childhood, I have been using local, our own varieties of maize.

[Question: why don’t you use the Chinese [hybrid] maize seed?]

I don’t know how to proper plant and take care of it. We are accustomed to planting the local varieties.

(28/04/2023)

Why has the transition to hybrid maize been so significant? First, the state’s role in agricultural extension has increasingly been taken over by the private sector. This transition has also been promoted by foreign actors such as the World Bank and USAID, as noted above. Seed dealers and agro-shop owners often play an important role in advising farmers on sowing decisions. Some of the seed shops visited by Spies had an own trial plot to test seed and demonstrate results to farmers. A salient characteristic with respect to hybrid maize is the prominent role of Chinese varieties, as noted above. Compared with other hybrid maize varieties, Chinese seed is relatively cheap and is widely offered in local shops. Chinese seed companies collaborate with local shop owners to market their seed in the country, making use of good bilateral relations and logistics channels between the two countries. Thus, Chinese companies benefit from the international development organisations which have actively encouraged private sector involvement and the commercialisation of agriculture.

Second, the transition to hybrid maize has been a self-reinforcing process, as a result of market dynamics. The rapid spread of hybrid maize varieties has increasingly limited farmers’ seed choices. Hybrid maize has pushed OPVs out of the market. As a result, farmers’ range of options has shrunk. In fact, local OPVs have almost disappeared. Indeed, only one of the more than 20 seed shops visited by Spies offered a local OP maize variety. Tajik state institutions still actively engage in breeding non-hybrid maize varieties, but these varieties do not become widely available to farmers. Farmers often mentioned this when asked about the reasons about the popularity of hybrids: “You cannot find local seeds here anymore. Local seeds almost disappeared” (farmer, 24 March 2023). Another farmer expressed (31 March 2023):

If we can find a way to use local seeds, improve breeding, then it would be better. If suitable local seed is available, would be better! Because of the quality. I also think that foreign hybrid seeds are not good, we are losing local varieties.

A third factor explaining the rapid proliferation of hybrid maize is related to intrinsic biological characteristics of maize. Reportedly, there are also hardly any farmers engaging in seed saving practices any longer, which is related to these characteristics. Maize is a highly cross-pollinating plant, and the fact that the vast majority of farmers use hybrid varieties makes it very difficult for others to maintain purity of their local variety, as a senior breeder specialised in maize explained (19 April 2023). When a farmer’s field is surrounded by fields sown with hybrid maize, the likelihood of the harvested grain containing genetic material from hybrid maize is high. This significantly constrains seed saving practices and thus contributes to the disappearance of local varieties. This specific characteristic of maize addresses the importance to attend to plants’ and seeds “capacity to act” (cf. Hebinck 2001).

As a result, many farmers have little choice but to cultivate hybrid maize. As a farmer responded when being asked whether everyone in his community is happy about the new hybrid maize seeds: “They have no other option, so they just use it!” (24 March 2023). Still, given that hybrid varieties perform well in terms of yield, the limited choice does not appear to be a major concern for most farmers.

Thus, farmers decisions are structured by specific opportunities and constraints. They try to maximise yields, for which limited options are available, namely costly hybrid seed. Given that only a relatively small share of land is allocated to maize, farmers consider the investment costs acceptable. Their choice expresses a trade-off between dependencies and profits. It is a pragmatic vision of progress. How do cotton seed choices compare? It is a political crop that features more complex production politics. This is discussed next.

### Stability and gradualism: a relatively closed cotton seed system

Cotton is Tajikistan’s primary agricultural export commodity. As a result, the crop is very important for the state, as well as the country’s ruling elites. Many farmers are pressured to allocate land to the crop, and there are also other ways in which the state incentivises cotton production. For instance, tax for land under cotton has been 50 per cent lower than for other crops (Hofman and Visser 2021).

In a context in which land is state-owned, pressure to grow cotton cannot easily be ignored. Some farmers with political or financial capital may negotiate cropping patterns, but growing cotton can be important for security of tenure, and also to convey loyalty to the state. In fieldwork, Hofman interacted with various farmers who stated that cotton was not a profitable crop. Cotton farm gate prices have fluctuated considerably in recent years. For instance, prices doubled between 2020 and 2021, but declined again after. Many farmers lament that there is no competition between cotton ginneries, and Hofman often recorded questions about prices offered. However, by growing cotton on a part of their land, farmers can produce other, more profitable or important crops, such as maize, on remaining parts, or use land for grazing animals. As such, growing cotton is a compromise for some farmers. Among these farmers, there is little interest to invest in quality cotton seed. This is different for relatively larger farms (above five hectares allocated to cotton), who specialise in cotton farming (see also Verweij-Novikova et al. 2024).

However, state pressure is not the only reason why farmers plant cotton. The fact that cotton is less perishable than many food crops is a reason why some farmers prefer cotton over food crops. Temperatures can exceed 40 to even 50 degrees Celsius in high summer in lowland Tajikistan. The rural infrastructure is in poor condition in many parts of the country, and many farmers have difficulties to transport and store (perishable) food crops. Hence, there are various factors that shape farmers’ cropping decisions and thus, also seed choices.

The cotton seed system has undergone various changes over the last three decades. These particularly relate to the role of the state and market characteristics. The Tajik state introduced a cotton seed import ban in the late 1990s that was only lifted in 2008 (on the ban, see Muminjanov et al. 2008). This happened when declining yields and debt among cotton farmers had reached a climax, and the Tajik state was under pressure from international donors to (more genuinely) liberalise the cotton sector (see also Hofman 2018). The Tajik state itself considered the lifting of the ban as a means to recover yields (see also Hofman, 2024). “We had no other solution,” the then-Minister of Agriculture said in an interview (5 May 2021, see also Hofman 2024). Improved seed was seen as an apolitical technical (and pragmatic) fix to increase yields.

The lifting of the ban brought a change in the range of actors involved in the socio-technical regime related to cotton. On paper, it granted farmers more autonomy in choosing cotton seed. However, it has not meant a radical change in the seed they sow: open pollinated varieties dominate, and little improved seed is imported and sown. Major transnational seed businesses involved in the international cotton seed market (e.g., Bayer) have not entered the country (Hofman 2024). At the same time, however, there has been foreign involvement in other ways. First, a number of foreign development organisations and companies operate in the cotton sector. There are a few Chinese agribusinesses with a physical presence in Tajikistan, engaged in selling cotton seed alongside cotton production and processing (Hofman 2024). A small number of other foreign actors (a few companies and organisations, such as the German GIZ and the Swiss Helvetas, and a British company) are mainly engaged with the introduction and market of certified cotton (e.g., Better Cotton Initiative and organic cotton). They provide extension services and sell cotton seed. Yet their initiatives are relatively small, and as is the case with all foreign actors operating in Tajikistan's rural economy, their activities are confined to specific localities, depending on state and elites’ approval. As a result, these donor projects risk exacerbating inequalities within the farming population in terms of access to extension and quality inputs (including seed), and thus the potential profitability of farm enterprises (see also Verweij-Novikova et al. 2024). The fact that state approval is required for international actors to enter specific localities testifies to the strong role of the state in the cotton sector and its seed regime, marking a stark contrast with maize.

Second, in addition to these donor-driven and corporate initiatives, foreign cotton seed companies and the international market are indirectly involved as some affluent farmers, private traders, and the Tajik state, import cotton seed, for instance from Uzbekistan, Turkey, and China. However, cotton seed imports remain limited; according to some specialists, they have accounted for less than five per cent of annually sown seed (see Hofman 2024). Some of the imported varieties enter the local circuit after a first season through reproduction by some farmers who ask ginneries to isolate seed when they sell their seed cotton, and by specially designated seed breeding farms. Yet, the relatively newly introduced seed is not accessible to all farmers. Hofman met small-scale farmers who lamented that seed imported and distributed by the state and international development organisations only spreads among well-connected farmers, remaining unavailable to the less capitalised ones. Indeed, attention to social differentiation is relevant for understanding cotton seed choice, much more than for maize.

The majority of farmers purchase OPVs from nearby cotton ginneries. These OPVs, including those bred by local breeders, have gradually entered the market over the years. Thus, the types of cotton and maize seed sown differ significantly. Also in terms of traders and sales points, as well as in the characteristics of extension agents, there is a contrast: cotton seed is rarely sold in small agro-shops (the primary place for maize seed sales), and instead of private seed dealers, most cotton farmers consult senior agronomists or neighbouring farmers for cotton seed advice. State officials and agricultural scientists occasionally also recommend new local varieties, bred by Tajik breeders. However, during fieldwork, Hofman observed that farmers had difficulties obtaining those varieties.

Two factors explain the popularity of ginneries as an outlet for seed. First, some farmers are contracted by a ginnery, which advances seed at the beginning of the growing season. Second, farmers choose to buy seed from ginneries due to the ease of acquiring and price: Seeds sold by ginneries are relatively cheap, about 25 per cent of the price of improved varieties (imported elite or so-called first-generation seed) (interview with a seed specialist, 30 October 2023; see also Hofman 2024). Areas sown with cotton tend to be relatively large and sowing rates are high. Farmers’ choice for ginnery seed reflects an attempt to limit seed cost, which contrasts with their choice for costly hybrid maize.

However, the low cost and ease of acquiring cotton seed come at a price. Farmers regularly lament that ginneries do not provide seed of homogenous variety, and they also question the “generation” of seed (see also Hofman 2024). Indeed, because the ginneries procure cotton from different farmers, the risk that seed varieties (of low quality) get mixed at ginneries is considerable. As an official stated (11 March 2021):

Imagine this is a ginnery floor. One farmer sells [the cotton variety] Buston, a second Oriyo, a third Iram. They all get mixed up before seeds are isolated for planting.

During fieldwork, Hofman regularly met farmers who referred to the name of the cotton variety they sowed with a sense of irony because ginneries cannot guarantee the purity of seeds. Many cotton farmers are also not able to identify the varieties in the field, lacking the knowledge to identify specific traits of the varieties. Hofman did not observe that concerns about pest resistance played a strong role in farmers’ seed choice. While cotton farmers regularly face pests, such as wilt and cotton spider mite, pests did not come to the fore in discussions on seed choice. Farmers rather apply various agro-chemicals, which are widely on offer in agroshops.

Yet, price and proximity alone lack explanatory power for farmers’ choice for locally available OPVs over improved varieties. As previously mentioned, improved seed is hard to come by, and farmers’ choice is given in by what the market offers. Furthermore, the incentive to invest in quality seed is low for many farmers: cotton farm gate prices are determined by ginneries purely based on weight. Ginneries check for debris, but do not focus on fibre length or strength (see also Hofman 2024), discouraging farmers to learn about specific traits of, and invest in, improved varieties.

In contrast to varieties’ traits and pest concerns, technology and the physical characteristics of cotton seed do play important roles in farmers’ cotton seed choice. One factor stands out: the technology of seed planters, and the technology used at cotton gins. Tajik farmers’ seed planters are designed for sowing linted seed, as the local ginneries only sell this type of seed. As such, the technology structures farmers’ range of options with regard to seed choices: Many cannot easily sow improved, delinted seed. Only after seed has entered the local circuit, it becomes available to those farmers. There are, however, a few capitalised farmers and companies owning seed planters suitable for delinted seed, and some skilled farmers sow imported, high quality delinted seed on small areas of land manually, after which they reuse the seed on their own fields (interview 6 September 2023). Yet few farmers do so; many farmers cannot afford such investments and lack the knowledge and ability, or interest and willingness, to engage in it. The dominance of linted seed and the way in which it limits farmers’ choices makes explicit how certain sets of rules and infrastructures over the course of time “order[…] the ‘material’” (Ploeg, van der et al. 2004, 4; see also Hebinck 2001), and how, in turn, the material orders farmers’ choices and practices.

The issue of lint also affects the interest in hybrid cotton seed, which is seldom sown in Tajikistan. Hybrid cotton seed was introduced by a Chinese company in the mid 2010s, but its attempts to market hybrid cotton seed did not last long, arguably because sales disappointed. Reportedly, some farmers had also started to reproduce the company’s hybrid variety (as OPV) as they did not observe declining yields (Hofman 2024). Yet probably the most important factor inhibiting the proliferation of hybrid cotton seeds is price: According to specialists, they are about eight to ten times more expensive than locally used OPVs (interview seed specialist, 30 October 2023). The costs would partially be offset by low sowing rates and yield gains, although the latter are unlikely as pronounced as in the case of hybrid maize. While the above-mentioned Chinese company reported yield gains of 50 to 100 per cent (five to six tons of seed cotton per hectare as opposed to yields reported by farmers, which ranged between two and three tons per hectare), during fieldwork Hofman regularly recorded doubt among farmers. Farmers did not question yield claims of the Chinese company but questioned their own ability to attain such yields in a context of outdated infrastructure and technology as well as financial constraints.

Price is also why state officials argue against hybrid cotton: “Our farmers are not able to [purchase hybrid varieties each year anew!] (state official, 24 October 2023, see also Hofman 2024). However, this argument is not valid given cotton seeds’ agronomic properties. Farmers also have to buy OPVs on a yearly basis as they cannot easily reproduce their own cotton seed. Most of them lack the technology to separate large volumes of seed from fibre, and if they were to process the seed manually, they would end up with fibre that they cannot easily sell as local ginneries would not accept it.^9^ Thus, farmers are required to buy cotton seed every year anew, regardless of the type of seed. Having said that, the interviewed official’s attitude towards hybrid cotton starkly contrasts with the state’s openness towards hybrid maize seed and other (food) crop seeds. It illustrates the importance to attend to the political nature of crops in understanding the commodification of seed.

Thus, whilst cotton is a cash crop produced for the export market, it is tightly controlled by the state and ruling elites in Tajikistan. Many farmers are not orientated towards the highest yielding, improved seed varieties. Their decisions do not reflect a reluctance for foreign varieties or concerns about seed autonomy, nor a rejection of a productivist ideology. Instead, farmers’ financial constraints and production imperatives affect their seed choices. As explained, for many farmers, cotton is not their priority. Their choice for reasonable and affordable seed is, at least for some, a compromise.

## Discussion

In this article, we have examined farmers’ maize and cotton seed choices in the complex context of Tajikistan, with a focus on the factors and actors affecting their choices, the strategies pursued by farmers, and what these strategies tell us about farmers’ notions of agricultural progress.

Our analysis illuminated a stark contrast between the maize and cotton seed systems, even though farmers are situated within one and the same landscape characterised by a productivist paradigm of agricultural development. The differences observed resulted from different constellations and dynamics within the socio-technical regimes specific to cotton and maize seed. Thus, we observed co-existing socio-technical (seed) regimes. We also illuminated a contrast with regard to differentiation within the population of cotton and maize growing farmers. Whereas social and political capital appears to influence farmers’ cotton seed choices, we did not observe this correlation for maize seed, where all farmers seemed to be caught up in similar twists and turns.

How can we make sense of the choices of farmers? In our analysis, we built on and adapted Geels’ (2011; 2019) framework of the socio-technical regime, enabling us to consider various actors and dimensions affecting farmers’ seed choices, specific to the context of Tajikistan. In what follows, we analyse the various actors and forces that shape farmers’ choices through this lens of the socio-technical regime. Geels (2011) identified five subregimes that comprise the socio-technical regime: the policy; science; technology; market; and socio-cultural regime. Many of the dynamics we observed and discussed in our article are not confined to one subregime alone. Seed decisions are shaped by various entangled factors: known yield gaps; production imperatives; technology; and the commodification of crops.

### The policy and science regimes

The Tajik state plays a strong role in the rural economy in various ways. For instance, it intervenes in land use and cropping patterns, and it steers and directs research undertaken by agronomists and seed breeders affiliated with domestic, public, institutes. The state’s interest in specific crops, such as cotton, has a bearing on the liberalisation of the market (and thus, market power and the relative commodification of seed). Thus, the role of the state is not always directly visible.

Indeed, the agrarian political economy has a strong bearing on market characteristics, both with regard to the seed market (discussed below under the “market regime”) as well as the outlets for seed cotton and maize (as a grain). Both maize and cotton are produced for the market, but the nature of the market and the imperatives of production differ. The contrast is stark. Cotton is produced for the global market, but many farmers do not grow the crop entirely voluntarily, and some hold that farm gate prices are suppressed and manipulated by ginneries. This is different for maize: Farmers can relatively autonomously produce and sell the crop on the local market. Thus, we contend that the crop-specific politics of production and marketing possibilities shape the willingness to take risks and invest in relatively expensive, improved seed. As Sinha (2022) contends, drawing on Scott (2017), crops are political. Farmers seed choices (relatively expensive yet high yielding maize varieties versus relatively cheap cotton seed) reflect their priority. To an extent, this might also be read as an expression of passive resistance to the top-down pressure to produce cotton. We shed light on a contrast between cotton and maize with regard to state involvement: a laissez faire approach regarding maize versus a stronghold on the cotton sector. These attitudes shape the development of the seed market in diverging ways. As Flachs and Stone (2019, 615) stated: “Farmers are not inherently risk-seekers or risk-avoiders but learn [and decide] in a dynamic context defined by different seeds and commodification in a larger political economy.”

### The market regime

Indeed, the commodification of cotton and maize seed differ, and market actors, including private and public institutions, actively contribute to shape seed decisions, albeit in very different ways. Whereas in the maize seed market, private domestic and international actors are the primary actors trading or selling seed, these actors do not play a significant role in the cotton seed sector. The significant difference in commodification is the result of the state’s role in agriculture and the political nature of cotton, which exemplifies the importance to attend to the interaction and overlap between the subregimes within the socio-technical regime, here related to policy and markets.

With a growing demand for and supply of hybrid maize seed, the market has become a structuring force in itself, as it has narrowed down farmers’ choices. Indeed, the range of options has been shrinking, as observed in many other cases worldwide where commodified seed has replaced local varieties (Montenegro de Wit 2016; Amanor 2024). In contrast, in Tajikistan’s cotton seed market the state has retained a relatively important role. This is not to say that the state has banned private sector involvement or sought to limit it: Given its interest in safeguarding cotton production, it has involved international donors to help securing access to affordable seed. However, the private sector does not play a significant role, and farmers are confronted with a paucity of alternatives: Hybrid and other improved cotton varieties are hardly offered on the local market. Where they are offered, they are, for many farmers, not affordable or not worth investing in.

With regard to the market regime, we have also shed light on the role of crop outlets themselves, which set production standards. In Tajikistan, for both maize and cotton, quality concerns, that is, fibre length, strength or colour for cotton, and cob characteristics for maize, play little to no role. High(er) quality produce does not translate into higher revenues, in simple terms: quantity trumps quality. As a result, farmers are not incentivised to invest in specific higher quality varieties.

### Technological regime and political ecology

Incorporating a political ecology lens in our analysis has not revealed a strong role of environmental factors such as water and crop pests in conditioning seed choices. However, we observed and illuminated the importance of crop properties and related technologies. We have highlighted this in three respects – one related to maize, two to cotton.

For one, in the case of maize, the highly cross-pollinating character of the plant acts as a biological force, that has challenged farmers to protect and maintain local OPVs. In a setting where hybrids have come to dominate, this characteristic of maize has constrained farmers wanting to plant OPVs. As a result, the growing use of hybrids, that was put in motion by market actors, has been accelerated. Concerning cotton, we observed two other material aspects that shape farmers’ seed choices. Firstly, the fact that their seed planters do not allow them to (efficiently) sow delinted seed. Secondly, the characteristic of the cotton crop itself. Farmers wanting to reproduce their seed autonomously (without being served by large ginneries) would have to engage in laborious manual labour to separate the seed from fibre. Thus, farmers’ manoeuvring space is not only structured by the rules set by individual (human) and institutional actors, but also by the material properties of crops and related technologies. This finding points to the importance of attending to materialities, including what may be called “crop agency”, in shaping sowing decisions and seed systems (cf. Fischer 2022; Fischer et al. 2022).

### Seed concerns? The socio-cultural regime

Where are quality, origin, and concerns about seed sovereignty, which feature so prominently in the international seed debate (Adhikari 2014; Gliessman 2023; Hernández Rodríguez 2023)? We could also ask: Do farmers seek to resist the dominant forces shaping the seed regime? While we did not ask explicit questions regarding seed sovereignty and autonomy, our contextual knowledge and conversations with farmers did not point to pronounced concerns, with the exception of a recent trend with regard to cotton seed: some farmers seek to autonomously reproduce seed. Yet this may be related to ginneries’ inadequate services, rather than resistance towards market dependency per se. More generally, we observed that, instead of attempts to limit dependency on off-farm or market actors, farmers rather conveyed an interest in affordable, foreign seed.

Thus, while Tajikistan is immensely rich in agrobiodiversity (particularly the mountainous parts and the Ferghana Valley), our interlocutors, engaged in the commercial production of maize and cotton, neither conveyed concerns about nor exhibited a desire to preserve local cultivars of these two crops, nor did they express concerns about a dependency on the market. As noted, yield and seed quality were much more important. Interestingly, explicit requests were often not limited to maize or cotton alone: During fieldwork, we often received requests for a wide range of high yielding food crop seeds.

To what extent can and do farmers’ choices give insight into their visions of agricultural progress, and how do farmers’ strategies articulate with paradigms about crop seeds and related pathways of agricultural development? Farmers’ seed decisions may in part be shaped by what Schmook et al. (2023) refer to as constrained imaginaries, that is, by ideas of desired pathways that are constrained by material realities as well as dominant paradigms. However, how seed choices and related strategies materialise complicates the picture: In the case of maize, farmers showcase a productivist strategy of using high-yielding hybrid seed; while in the case of cotton, farmers’ seed choices suggest a different strategy, as they primarily sow local OPVs of, what could be called, mediocre quality. In this sense, farmers pursue strategies that challenge any binary notion of two distinct pathways associated with local versus improved seed that the international seed debate suggests.

## Conclusion

In this article, we analysed the set of actors and factors affecting farmers´ seed choices. Our insights contribute to the literature on farming pathways and practices in two ways.

For one, we addressed the complexity of seed choice, by attending to the market; the state; production politics; technology and, seed’s intrinsic material properties. By considering production politics, we also highlighted how seed choice can offer a lens through which to analyse and understand the political economy of farming. Attention to the political nature of crops (Sinha 2022; Fischer et al. 2022) appeared particularly apposite for the study of maize and cotton seed decisions in Tajikistan, where the state has a strong role in agriculture.

We observed and addressed salient differences in the politics of production between maize and cotton, which have a bearing on the commodification of seed, and seed choice. Thus, we situated farmers’ seed decisions in the socioeconomic and sociopolitical context in which they produce cotton and maize. We shed light on the “variable importance placed on local variation, profit, and farmer knowledge” (cf. Flachs and Stone 2019, 617). As a corollary, we noticed that farmers’ability to learn and gain experiential knowledge about seed differed. Indeed, “different seeds can lead to very different types of learning and decision-making” (Flachs and Stone 2019, 615).

Second, our findings revealed that seed choice does not correlate to specific imaginaries. Our interlocutors exhibited a notion of what we call pragmatic rather than paradigmatic progress. Different attitudes towards seed co-exist, which may appear contradictory at first sight but can be explained by the intricacies of the local context. Thus, our findings challenge the dualistic notions of rural and agrarian development, with a dichotomy between extensive, agroecological farming on the one hand, and highly productivist, what some call “modern,” models of farming, on the other. We contend that farmers’ struggles and stances with regard to seed should be taken more seriously in discussions of potential and preferred agrarian pathways. In the words of Montenegro de Wit (2016, 632), “what occurs on the ground is far more eclectic.” Instead of paradigms, contextual characteristics (“on the ground”) structure farmers’ decisions.

We identify several avenues for future research. Firstly, future research could examine seed choice with specific attention to differences across strata of farmers, with regard to farm size, off-farm capital, and gender. Secondly, more research on seed choice with regard to food crops grown on household plots is warranted. Thirdly, analysis could focus on Tajik farmers and rural households in localities with different geographic and ecological characteristics, such as Tajikistan’s mountainous areas, where people have experienced less radical overhauls in access to land in the Soviet period. Lastly, skilling and knowledge encounters, with regard to seed and seed regime changes, merit more attention.

While the case of Tajikistan is unique in its Soviet legacy and in the politics of production that particularly concerns cotton, our findings speak to dynamics beyond the case of Tajikistan or Central Asia alone. Our insights call for more nuanced approaches and understandings of farming and farmers’ decision-making. Thus, we concur with Luna (2020), who addressed the importance of contextualising seed choice and challenging essentialist notions of different types of agricultural producers, and draw attention to the ways in which farmers’ ideas and imaginaries of specific agrarian trajectories are constrained (cf. Schmook et al. 2023). Our findings also resonate with observations by Arora et al. (2024, 1) who examined industrial versus agroecological pathways of rice seed use in India, where “an overall picture in support of one pathway did not emerge.” Tajik farmers’ cotton and maize seed decisions reflect what farmers “[perceive to be] materially possible” (Schmook et al. 2023, 306). Farmers’ practices do not neatly align with grand paradigms. Instead, their choices exhibit a localised vision of progress that should inform discussions and debates about the way forward.

## Footnotes

1 In this article, we use the notion of “improved seed” to refer to, not only, hybrid and genetically modified seeds, but more generally to seeds developed through advanced breeding practices in the “purview of off-farm experts” (Mueller and Flachs 2022, 461). In this definition, “improved seed” also includes the high-yielding wheat and rice varieties introduced with the Green Revolution in the 1960s to 1980s (Byerlee and Siddique 1994; Glaeser 2010).

2 Furthermore, maize and cotton are global commodity crops, of which “improved” varieties have proliferated rapidly across the globe in recent decades, with manifold consequences on seed systems (Luna 2020; on discussions see, for instance, Curry 2023; Zuberi et al. 2024).

3 While we occasionally refer to the socio-technical regimes that we investigate as “seed regimes”, our use of the notion of seed regime is somewhat different from the seed regime concept as outlined by Kuyek (2007) and recently adopted by some authors (Lyon et al. 2021; Hernández Rodríguez 2023; Spies 2025). The latter draw on the well-established notion of food regime (McMichael 2009; Otero 2016) to analyse the governance and power structures dominating a seed system in a given period of time.

4 In our analysis, we narrowed down the “user and market subregime” to market regime.

5 Households have enjoyed autonomy in their use of household plots. The practices and seed choices for the production of (food) crops on household plots is beyond the scope of our analysis.

6 *Dehqon* farms (defined later in the text) can obtain inheritable land use rights. For other types of producers, such as limited liability companies, agricultural enterprises, and foreign corporations, access to land is limited to 20–50 years (with possibility of extension).

7 In our analysis, we draw primarily on qualitative findings and data. Official statistics as well as farms’ legal status conceal more than they reveal. For instance, there are large farms with individual shareholders who are all registered as individual farmers but in practice merely act as labourers of the large farm; and, producer cooperatives that act like small-scale *dehqon* farms. This diversity and lack of accurate data complicate an analysis of the correlation between farm size, farm ownership and seed choice.

8 A feature unique to Tajikistan is that many villages in the lowland areas of the Khatlon region were established in the Soviet period. This involved the resettlement of mountainous dwellers, a process that uprooted people and disrupted people’s ties to land and their ecological knowledge.

9 A salient development in this regard is the emergence of small “mini-ginneries” (see also Hofman 2024) which enables farmers to process their own cotton seed, sell the seed to other farmers in a later season, and sell the fibre on the domestic market. Research on this dynamic is yet to be undertaken.

## References

[CR1] Adhikari, Jagannath. 2014. Seed sovereignty: Analysing the debate on hybrid seeds and GMOs and bringing about sustainability in agricultural development. *Journal of Forest and Livelihood* 12:33–46.

[CR2] Almekinders, Conny, J. M., Michael Koen Beumer, Michael Hauser, Marcel Misiko, Agnes O Gatto, Nkurumwa, and Olaf Erenstein. 2019. Understanding the relations between farmers’ seed demand and research methods: The challenge to do better. *Outlook on Agriculture* 48:16–21. 10.1177/0030727019827028

[CR3] Amanor, Kojo S. 2024. Contradictions between commercializing seeds, empowering smallholders farmers, and promoting biodiversity in ghana: Seed policy within a historical framework. *Elem Sci Anth* 12:00004. 10.1525/elementa.2023.00004

[CR4] Arora, Saurabh, Bhuvana Narayanarao, Nimisha Mittal, and Vadekkal Rasheed Sulaiman. 2024. Agricultural innovations for sustainability? Diverse pathways and plural perspectives on rice seeds in Odisha, India. *Agriculture and Human Values* 42:1155–1172. 10.1007/s10460-024-10666-0 10.1007/s10460-024-10666-0 PMC12098188 40417319

[CR66] Byerlee, Derek, and Akmal Siddiq. 1994. Has the Green Revolution been sustained? The quantitative impact of the seed-fertilizer revolution in Pakistan revisited. *World Development* 22:1345–1361. 10.1016/0305-750X(94)90008-63.

[CR5] Clapp, Jennifer. 2021. The problem with growing corporate concentration and power in the global food system. *Nature Food* 2:404–408. 10.1038/s43016-021-00297-7 37118223 10.1038/s43016-021-00297-7

[CR6] Curry, Helen Anne. 2023. Breeding confusion: Hybrid seeds and histories of agriculture. *The Journal of Peasant Studies* 50:1037–1055. 10.1080/03066150.2023.2180357 37346474 10.1080/03066150.2023.2180357 PMC10281510

[CR7] ETC Group. 2022. *Food barons 2022: crisis profiteering, digitalization and shifting power*.

[CR8] Fischer, Klara. 2022. Why Africa’s new green revolution is failing – Maize as a commodity and anti-commodity in South Africa. *Geoforum* 130:96–104. 10.1016/j.geoforum.2021.08.001

[CR9] Fischer, Klara, Jostein Jakobsen, T. Ola, and Westengen. 2022. The political ecology of crops: From seed to state and capital. *Geoforum* 130:92–95. 10.1016/j.geoforum.2021.12.011

[CR52] Flachs, Andrew, José Becerra, Fionna Fahey, and Patricia Mathu. 2025. Skilling and social reproduction. *Outlook on Agriculture* 54:150–161. 10.1177/00307270251326606

[CR10] Flachs, Andrew, and Stone Glenn Davis. 2019. Farmer knowledge across the commodification spectrum: Rice, cotton, and vegetables in Telangana, India. *Journal of Agrarian Change* 19:614–634. 10.1111/joac.12295

[CR11] Geels, Frank W. 2011. The multi-level perspective on sustainability transitions: Responses to seven criticisms. *Environmental Innovation and Societal Transitions* 1:24–40. 10.1016/j.eist.2011.02.002

[CR12] Geels, Frank W. 2019. Socio-technical transitions to sustainability: a review of criticisms and elaborations of the multi-level perspective. *Current Opinion in Environmental Sustainability* 39:187–201. 10.1016/j.cosust.2019.06.009

[CR13] Geels, Frank W., Johan Schot. 2007. Typology of sociotechnical transition pathways. *Research Policy* 36:399–417. 10.1016/j.respol.2007.01.003

[CR77] Glaeser, Bernhard, ed. 2010. The Green Revolution revisited: Critique and alternatives. Oxon: Routledge.

[CR14] Gliessman, Steve. 2023. The stories of seed sovereignty. *Agroecology and Sustainable Food Systems* 47:643–645. 10.1080/21683565.2023.2182526

[CR15] Hayward, Daniel, Irna Hofman, and Kramer Gillin. 2022. Tajikistan - context and land governance. *Land Portal*.

[CR16] Hebinck, Paul. 2001. Maize and socio-technical regimes. In *Resonances and dissonances in development: actors, networks and cultural reportoires*, ed. Paul Hebinck, and Gerard Verschoor. 119–139. Assen: Van Gorcum.

[CR17] Hernández Rodríguez, Carol. 2023. Seed sovereignty as decommodification: A perspective from subsistence peasant communities in Southern Mexico. *The Journal of Peasant Studies* 50:986–1013. 10.1080/03066150.2022.2025780

[CR55] Hofman, Irna. 2018. Soft budgets and elastic debt: farm liabilities in the agrarian political economy of post-Soviet Tajikistan. *The Journal of Peasant Studies* 45:1360–1381. 10.1080/03066150.2017.1293047

[CR56] Hofman, Irna. 2021. Migration, crop diversification, and adverse incorporation: understanding the repertoire of contention in rural Tajikistan. *Canadian Journal of Development Studies / Revue Canadienne d’études Du Développement* 42: 499–518. 10.1080/02255189.2020.1788519

[CR50] Hofman, Irna. 2024. Seeds of empire or seeds of friendship? The politics of the diffusion of Chinese cotton seeds in Tajikistan. *Journal of Agrarian Change* 24:e12581. 10.1111/joac.12581

[CR51] Hofman, Irna, and Oane Visser. 2021. Towards a geography of window dressing and benign neglect: The state, donors and elites in Tajikistan’s trajectories of post-Soviet agrarian change. *Land Use Policy* 111:105461. 10.1016/j.landusepol.2021.105461

[CR18] Kilwinger, Fleur BM, Anne M Pricilla Marimo, J. M. Rietveld, Conny, Almekinders, and Ynte K van Dam. 2020. Not only the seed matters: Farmers’ perceptions of sources for banana planting materials in Uganda. *Outlook on Agriculture* 49: 119–132. 10.1177/0030727020930731 10.1177/0030727020930731 PMC7684323 33281230

[CR19] Kloppenburg, Jack Ralph. 2004. *First the seed: the political economy of plant biotechnology, 1492–2000.* 2nd ed. Science and technology in society. Madison, Wis: University of Wisconsin Press.

[CR20] Kuyek, Devlin. 2007. Sowing the seeds of corporate agriculture: The rise of canada’s third seed regime. *Studies in Political Economy* 80:31–54. 10.1080/19187033.2007.11675082

[CR21] Lawhon, Mary, James T., and Murphy. 2012. Socio-technical regimes and sustainability transitions: Insights from political ecology. *Progress in Human Geography* 36:354–378. 10.1177/0309132511427960

[CR22] Lerman, Zvi, and David Sedik. 2018. Transition to smallholder agriculture in central Asia. *Journal of Agrarian Change* 18:904–912. 10.1111/joac.12282

[CR23] Levidow, Les, Michel Pimbert, and Gaetan Vanloqueren. 2014. Agroecological research: Conforming—or transforming the dominant agro-food regime? *Agroecology and Sustainable Food Systems * 38:1127–1155. 10.1080/21683565.2014.951459

[CR24] Luby, Claire H., and Irwin L. Goldman. 2016. Freeing crop genetics through the open source seed initiative. *PLOS Biology* 14:e1002441. 10.1371/journal.pbio.1002441 27093567 10.1371/journal.pbio.1002441 PMC4836679

[CR25] Luna, Jessie K. 2020. Peasant essentialism in GMO debates: Bt cotton in Burkina Faso. *Journal of Agrarian Change* 20:579–597. 10.1111/joac.12381

[CR26] Lyon, Alexandra, Harriet Friedmann, and Hannah Wittman. 2021. Can public universities play a role in fostering seed sovereignty? *Elementa: Science of the Anthropocene* 9:00089. 10.1525/elementa.2021.00089

[CR27] McMichael, Philip. 2009. A food regime genealogy. *The Journal of Peasant Studies* 36:139–169. 10.1080/03066150902820354

[CR28] Montenegro de Wit, Maywa. 2016. Are we losing diversity? Navigating ecological, political, and epistemic dimensions of agrobiodiversity conservation. *Agriculture and Human Values* 33:625–640. 10.1007/s10460-015-9642-7

[CR29] Mueller, Natalie G., Andrew Flachs. 2022. Domestication, crop breeding, and genetic modification are fundamentally different processes: Implications for seed sovereignty and agrobiodiversity. *Agriculture and Human Values* 39:455–472. 10.1007/s10460-021-10265-3

[CR30] Mukhamedova, Nozilakhon, and Kai Wegerich. 2018. The feminization of agriculture in post-Soviet Tajikistan. *Journal of Rural Studies* 57:128–139. 10.1016/j.jrurstud.2017.12.009

[CR333] Muminjanov, Hafiz, Anna Berlin, Rutger Persson, and Munira Otambekova. 2008. Focus on seed programs: The Tajikistan seed industry. Aleppo: ICARDA

[CR31] Nekbakhtshoev, Navruz, and Babu Suresh Chandra. 2022. Agrarian reform and water resource management: A case study and lessons from Tajikistan. *Central Asian Journal of Water Research* 8:1–26. 10.29258/CAJWR/2022-R1.v8-1/1-26.eng

[CR32] OECD. 2018. *Concentration in seed markets: potential effects and policy responses*. OECD. 10.1787/9789264308367-en.

[CR33] Otero, Gerardo. 2016. Review of Philip McMichael’s food regimes and agrarian questions. *Journal of World-Systems Research* 22:299–305. 10.5195/jwsr.2016.651

[CR34] Ploeg, van der Jan, Johan Douwe, Arie Bouma, Frits Rip, Flaminia Rijkenberg, Ventura, S. C. Johannes, and Wiskerke. 2004. On regimes, novelties, niches and co-production. In *Seeds of transition*, 1–30. Assen: Royal Van Gorcum.

[CR35] Qaim, Matin. 2020. Role of new plant breeding technologies for food security and sustainable agricultural development. *Applied Economic Perspectives and Policy* 42:129–150. 10.1002/aepp.13044

[CR36] Rip, Arie, René, and Kemp. 1998. Technological change. In *Human choice and climate change*, 327–399. Columbus, Ohio: Battelle.

[CR37] Sandström, Emil, Tove Ortman, Christine A Watson, Jan Bengtsson, Clara Gustafsson, Göran, and Bergkvist. 2024. Saving, sharing and shaping landrace seeds in commons: Unravelling seed commoning norms for furthering agrobiodiversity. *Agriculture and Human Values* 41:1825–1840. 10.1007/s10460-024-10581-4

[CR38] Schapiro, Mark, and David Talbot. 2018. *Seeds of Resistance: The Fight to Save Our Food Supply*. 1st edition, 1st printing. New York: Hot Books.

[CR39] Schmook, Birgit, Claudia Radel, Lindsey Carte, and Richard L. Johnson. 2023. In the shadow of the Green Revolution: constrained spatial imaginaries and smallholder farming in Guatemala’s Pacific lowlands. *The Professional Geographer* 75:305–315. 10.1080/00330124.2021.2014907

[CR40] Scott, James C. 2017. *Against the grain: A deep history of the earliest States*. New Haven and London: Yale University Press.

[CR41] Sievers-Glotzbach, Stefanie, and Anja Christinck. 2021. Introduction to the symposium: seed as a commons—exploring innovative concepts and practices of governing seed and varieties. *Agriculture and Human Values * 38:499–507. 10.1007/s10460-020-10166-x

[CR42] Sinha, Shreya. 2022. From cotton to paddy: Political crops in the Indian Punjab. *Geoforum* 130:146–154. 10.1016/j.geoforum.2021.05.017

[CR53] Spies, Michael. 2025. Local seed systems and Global China: The spread of Chinese hybrid seeds in Pakistan and Tajikistan. *The Journal of Peasant Studies* 52:1295–1322. 10.1080/03066150.2025.2462762

[CR44] Taylor, Marcus. 2020. Hybrid realities: Making a new Green Revolution for rice in South India. *The Journal of Peasant Studies* 47:483–502. 10.1080/03066150.2019.1568246

[CR43] ter Steeg, Emily M. S., Paul C. Struik, and Richard G. F. Visser, Pim Lindhout. 2022. Crucial factors for the feasibility of commercial hybrid breeding in food crops. *Nature Plants* 8:463–473. 10.1038/s41477-022-01142-w 35513713 10.1038/s41477-022-01142-w

[CR45] Verweij-Novikova, Irina, Helena Farrall, Antonio Guerreiro de Brito, Irna Hofman, and Tahmina Sayfulloeva. 2024. *Value chain analysis of cotton in Tajikistan. Report for the European Union, DG-INTPA Value Chain Analysis for Development Project (VCA4D CTR 2017/392–416.* (unpublished report). Brussels: Agrinatura.

[CR46] Wilson, Geoff A., J. F. Rob, and Burton. 2015. Neo-productivist’ agriculture: Spatio-temporal versus structuralist perspectives. *Journal of Rural Studies* 38:52–64. 10.1016/j.jrurstud.2015.02.003

[CR47] Zakirova, Aksana, Henryk Alff, and Matthias Schmidt. 2023. Cash crop or food crop? Socioeconomic and geopolitical factors affecting smallholder farmer crop selection in times of crisis in Southwestern Tajikistan. *Frontiers in Agronomy* 5:1228165. 10.3389/fagro.2023.1228165

[CR49] Zuberi, Mehwish, Michael Spies, and Nielsen Jonas Østergaard. 2025. Constrained spatial imaginaries of smallholder farmers: perspectives from South Punjab, Pakistan. *Journal of Integrative Environmental Sciences* 22:2536263. 10.1080/1943815X.2025.2536263

[CR48] Zuberi, Mehwish, Michael Spies, Ø. Jonas, and Nielsen. 2024. Is there a future for smallholder farmers in bioeconomy? The case of ‘improved’ seeds in South Punjab, Pakistan. *Forest Policy and Economics* 158:103100. 10.1016/j.forpol.2023.103100

